# “I AM TOO EXHAUSTED TO BE PROACTIVE”: CHALLENGES IN NAVIGATING END-OF-LIFE CARE FOR PARENTS WITH DEMENTIA IN CHINA

**DOI:** 10.1093/geroni/igae098.2379

**Published:** 2024-12-31

**Authors:** Yifan Lou, Jinyu Liu, Kedong Ding

**Affiliations:** Virginia Commonwealth University, New Haven, Virginia, United States; Baylor University, Waco, Texas, United States; Case Western Reserve University, Cleveland, Ohio, United States

## Abstract

While hospice is rapidly developing in China, the services are mostly provided to and used by patients with cancer. The end-of-life experiences of persons living with dementia (PLWD) and their caregivers are largely overlooked in the current practice and literature. This study aims to explore unique challenges and stressors that family caregivers experience when navigating end-of-life care for PLWD. Interview data were collected from in-depth interviews with 17 caregivers of PLWD in China and 5 primary care doctors working with them in Shanghai, China. Hybrid grounded theory model was used to guide analysis. Five themes emerged from the data describing multi-level challenges and stressors guided by the social ecological theory. On the individual and interpersonal levels, emotional exhaustion of decision-making was most salient among PLWD caregivers, especially when there was lack of communication on required decision-making paperwork at emergency rooms. Caregivers also experienced decisional conflicts among siblings and uncertainty about the validity of PLWD’s expressed wishes at the advanced dementia stage. On the mezzo level, a common theme emerged around the stigma toward PLWD (as opposed to hospice for people with cancer) in the community, which prevents caregivers from seeking services and support for their dying loved ones. On the macro level, the lack of disease-specific end-of-life resources for PLWD, which results in long waiting lists and increasing service prices, was a major challenge for caregivers in finding care aligned with PLWD’s needs. Multi-level interventions are needed to address caregivers’ challenges in navigating end-of-life care for PLWD in China.

